# Interferon-γ Autoantibodies as Predisposing Factor for Nontuberculous Mycobacterial Infection

**DOI:** 10.3201/eid2206.151860

**Published:** 2016-06

**Authors:** Florent Valour, Thomas Perpoint, Agathe Sénéchal, Xiao-Fei Kong, Jacinta Bustamante, Tristan Ferry, Christian Chidiac, Florence Ader, on behalf of the Lyon TB study group

**Affiliations:** Hospices Civils de Lyon, International Center for Research in Infectiology, Université Claude Bernard Lyon 1, INSERM U1111, Lyon, France (F. Valour, T. Perpoint, A. Sénéchal, T. Ferry, C. Chidiac, F. Ader);; Necker-Enfants Malades Hospital, INSERM, Paris Descartes University, Paris, France (J. Bustamante);; St. Giles Laboratory of Human Genetics of Infectious Diseases, Rockefeller University, New York, New York, USA (X.-E. Kong, J. Bustamante)

**Keywords:** Nontuberculous mycobacteria, non-tuberculous, interferon-gamma, interferon-γ, antibody, autoantibodies, Herpesviridae, monoclonal, Interleukine 12, immunodeficiency, antimycobacteria, Mycobacterium, intracellulare, fortuitum, avium, Laos, tuberculosis and other mycobacteria, bacteria

**To the Editor:** Recent advances in the understanding of antimycobacterial immune response have led to descriptions of predisposing conditions to dissemination of nontuberculous mycobacteria (NTM) infection. The interferon- γ (IFN-γ)/interleukin-12 (IL-12) axis is a critical pathway for intracellular killing of mycobacteria (Technical Appendix Figure) (*1*).We report a case of disseminated NTM infection in a woman from Laos who showed IFN-γ autoantibodies. We also conducted a literature review to review similar cases.

A previously healthy 50-year-old woman from Laos was referred to Hospices Civils de Lyon, France, for fever and generalized lymphadenopathies; pathological examination revealed a nonnecrotizing granuloma. Culture yielded *Mycobacterium fortuitum*. After a 3-month azithromycin/ciprofloxacin regimen, symptoms resolved. A few months later, a computed tomography scan showed persistence of enlarged mediastinal lymph nodes; biopsy disclosed nonspecific sinus histiocytosis. Mycobacterial cultures were negative. One month later, the patient reported intense dorsal spine pain. She was diagnosed with multifocal vertebral osteomyelitis, and tissue specimens tested positive for *M. intracellulare*. Azithromycin/ciprofloxacin was prescribed again. At 6 months, lesions worsened, and epidural abscess led to spinal cord compression, requiring decompressive laminectomy. Postoperative cultures were positive for *M. intracellulare*. Treatment was changed to azithromycin, rifabutin, ethambutol, and amikacin for 2 weeks, then rifabutin and moxifloxacin, which led to clinical improvement. 

The patient experienced 2 episodes of thoracic herpes zoster concomitantly to each episode of illness. The recurrent NTM infection led us to investigate her immune status. Results of standard immunologic tests (leukocyte, serum level of immunoglobulin isotypes, and T-, B-, and NK0cell counts) and in vitro T-cell proliferation were normal; HIV testing was negative. Given its critical role in *Mycobacteria* clearance by host cells, we used IFN-γ whole-blood activation to investigate further. IFN-γ plasma level was undetectable, excluding complete forms of IFN-γ R1/R2. IL-12 p40 subunit plasmatic level was decreased, but the IL-12 receptor was present and functional. In addition, both BCG and IL-12 stimulations failed to trigger IFN-γ production. Ultimately, the detection of IFN-γ autoantibodies in high levels explained these abnormalities. Antimycobacterial treatment was continued for 2 years, then changed to azithromycin suppressive therapy; IFN-γ autoantibodies remained positive at the time of this report, 2 years after azithromycin initiation.

We reviewed the literature and found 63 other cases of IFN-γ autoantibody-related NTM infection with sufficient detail to provide clinically relevant material for optimizing disease management (Table; Technical Appendix). These cases overwhelmingly occurred among Asian populations (92.2%) (*2*). Disease incidence may be underestimated, as suggested by large studies describing Asian patients with disseminated NTM infection without evidence of impaired immunity but for whom the IL-12/IFN-γ axis was not investigated (*3*). This acquired autoantibodies–mediated immunodeficiency is more frequent among women, in whom the disease typically manifests in the second half of adult life (median 48 [IQR 44.8–60.0] years of age). Reported female:male sex ratios are 23/36 in Asian-born (n = 59) and 4/1 in non-Asian (n = 5) case-patients, respectively. Mechanism of the disease is briefly discussed in the Technical Appendix.

**Table:** Summary of clinically relevant characteristics of 64 case-patients with IFN-γ autoantibody-related nontuberculous mycobacterial infection found in literature review*

Characteristics	Value
Demographics	
Female sex	40 (62.5)
Median age, y (IQR)	48.0 (44.8–60.0)
Asian origin	59 (92.2)
Other autoimmune diseases	7/27 (25.9)
Etiologic agents	
* Mycobaterium avium*	23 (40.4)
* M. abscessus*	18 (31.6)
* M. fortuitum*	7 (12.3)
* M. tuberculosis*	*6 (10.5)*
Other opportunistic infections	39/52 (75.0)
*Herpesviridae* reactivations	23/52 (44.2)
*Salmonella* spp.	13/52 (25.0)
Median duration of intensive-phase therapy, mo (IQR)	31.0 (22.8–60.0)
≥6	31/31 (100)
≥12	29/31 (93.5)
≥18	27/31 (87.1)
≥24	23/31 (74.2)
Long-term antimicrobial suppressive therapy	3/30 (10.0)
Immunomodulatory therapies	10/30 (33.3)
Rituximab	6/30 (20.0)
IFN-γ	5/30 (16.7)
Intravenous immunoglobulins	2/30 (6.7)
Plasmapheresis	2/30 (6.7)
Cyclophosphamid	1/30 (3.3)
Outcome	
Cure	21/62 (33.9)
Improvement	6/62 (9.7)
Relapse/persistence	29/62 (46.8)
Death	6/62 (9.7)
*Values are no. (%) except as indicated. Only articles with sufficient detail were analyzed. A complete listing of articles reviewed from the literature is provided in the online Technical Appendix Table (http://wwwnc.cdc.gov/EID/article/22/6/15-1860-Techapp1.pdf). IQR, interquartile range; IFN-γ, interferon gamma. †Denominators indicate number of case-patients for which category of data was available.	

*M. avium* complex predominated in the literature review, accounting for 40.4% of cases, followed by *M. abscessus* (31.6%) and *M. fortuitum* (12.3%). Infections were mostly multifocal, affecting lymph nodes (n = 51, 79.7%), osteoarticular tissue (n = 37, 57.8%), lungs (n = 30, 46.9%), and skin and/or soft tissues (n = 24, 37.5%). Aside from NTM infections, other opportunistic infections were reported in 39 (75.0%) patients, mostly *Herpesviridae* reactivations (44.2%) and *Salmonella* infections (25.0%) (*3*–*6*).

Specific treatment for IFN-γ autoantibodies–associated NTM infection is not codified and required prolonged, multiple-drug regimens. The median treatment duration for the studies we reviewed was 31 (IQR 22.8–60.0) months. In some studies, clinicians used IFN-γ administration (5 patients, 1 of whom was cured), but treatment likely was invalidated by the autoimmune-driven inhibitory activity (*5*,*2*,*7*). Other strategies included intravenous immunoglobulin (n = 2), plasmapheresis (n = 2), and cyclophosphamide (n = 1) (*7*–*9*). The use of rituximab, a chimeric anti-CD20 monoclonal antibody targeting B-cells, has been recently associated with clinical response and decrease in IFN-γ autoantibody levels as well as neutralizing ability (*6**,**7*).

Final outcome was available for 56 patients who completed the intensive treatment phase; 21 (37.5%) were declared cured. Six (10.7%) patients died, and 29 (51.8%) had persistent or relapsing infections. At the time of this report, additional patients were still being treated and showed improvement of symptoms. Despite this high rate of failure, long-term antimicrobial drug suppressive therapy has rarely been proposed as a causal factor. The origin of the case we report was related to the use of azithromycin suppressive therapy, similarly to disseminated NTM disease prophylaxis in HIV-infected patients before the era of highly active antiretroviral therapies (*10*), assuming the risk/benefit balance including the possibility of NTM macrolide-resistant strain selection.

IFN-γ autoantibodies are evidence of acquired immunodeficiency that should be considered in cases of unexplained disseminated NTM infections in Asian-born persons. Use of immunomodulation strategies is still debated, and long-term suppressive treatment should be considered for persisting high levels of neutralizing antibodies.

Technical Appendix Discussion of mechanism of the disease, a supplemental illustration, and data concerning the interferon–gamma/interleukin-12 axis and factors supporting nontuberculous mycobacterial infection caused by interferon-γ autoantibodies.
