# Supplementary material for: Interferon-γ Autoantibodies as Predisposing Factor for Nontuberculous Mycobacterial Infection
Source: Emerg Infect Dis. 2016 Jun;22(6):1124–6. doi: 10.3201/eid2206.151860 (PMC4880074; doi:10.3201/eid2206.151860)
Supplement: Technical Appendix — Discussion of mechanism of the disease, a supplemental illustration, and data concerning the interferon–gamma/interleukin-12 axis and factors supporting nontuberculous mycobacterial infection caused by interferon-γ autoantibodies. [file 15-1860-Techapp-s1.pdf]

Article DOI: http://dx.doi.org/10/3201/eid2206.151860

Interferon-γ Autoantibodies as Predisposing Factor for Nontuberculous Mycobacterial Infection

Technical Appendix

The mechanism of disease for nontuberculous mycobacterial infection is not completely understood, but 3 likely components include 1) genetics, because a strong association with HLA-Drβ1602 and HLA-Drβ0502 was demonstrated among 17 patients, but without familial clustering (*1*); 2) impaired immune signaling leading to autoantibody production; and 3) environmental factors, possibly taking place during childhood, considering that most studied case-patients are Asian-born.

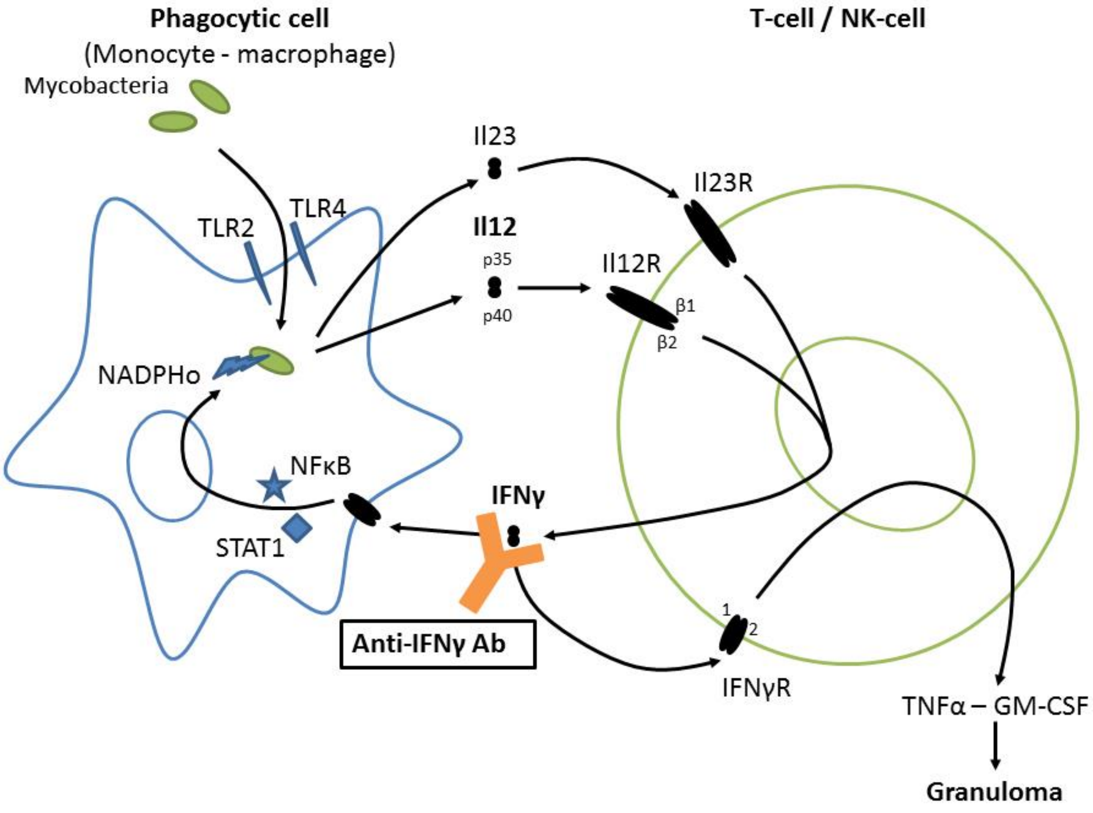

Technical Appendix Figure. The interferon-gamma (IFN-γ)/interleukine-12 (IL-12) axis as a critical pathway for intracellular killing of mycobacteria.

Technical Appendix Table. Published cases of non-tuberculous mycobacterial infection caused by interferon-γ autoantibodies, 2004–2015*

Case-patient sex, age (y)	Origin	Baseline treatment regimen	Duration (months)	Immune therapies	Outcome	Reference
M, 47	Philippines	AMK, CAM, EB, INH, PZA, doxycyline	NA	IFNg	Died	Doffinger et al., 2004 (*2*)
F, 25	Thailand	NA	NA	None	Died	Hoflich et al., 2004 (*3*)
F, 46	United Kingdom	CFZ, EB, CAM, RIB	42	IFNg	Cured	Kampmann et al., 2005 (*4*
M, 32	South Africa	EB, CAM, RIB	NA	None	Cured	(*4*)
F, 59	United Kingdom	NA	NA	None	Cured	(*4*)
F, 43	Taiwan	NA	60	None	Cured	Patel et al., 2005 (*5*)
F, 52	China	NA	> 60	None	Persistent infection	(*5*
F, 40	Philippines	NA	> 60	None	Persistent infection	(*5*)
F, 31	Philippines	NA	> 30	None	Persistent infection	(*5*)
F, 46	Philippines	CAM, EB, INH, LNZ, MFL, TIG	> 60	IFN-γ, rituximab	Relapse	(*5*)
F, 69	Philippines	AMK, AMX/CLV, AZI, CFX, EB, INH, LNZ, MER, RMP, TIG	> 84	IFN-γ, IvIg, plasmapheresis, rituximab	Relapse	Browne et al., 2012 (*6*)
M, 54	Japan	CAM, EB, RMP, SM	NA	None	Cured	Tanaka, 2007 (*7*)
F, 38	Philippines	AZI, EB, RIB	Under treatment	Cyclophosphamide, plasmapheresis	Improved	Baerlecken et al., 2009 (*8*)
F, 44	Japan	CAM, EB, MFL, RMP, SM	> 18	IvIg	Cured	Koya et al., 2009 (*9*)
M, 56	Thailand	EB, INH, MFL, PZA, RMP	24	None	Relapse	Kampitak et al., 2011 (*10*)
M, 45	Thailand	EB, INH, PZA, RMP	Under treatment	None	Improved	(*10*
M, 39	Thailand	EB, INH, MFL, PZA, RMP	Under treatment	None	Improved	(*10*)
F, 50	Laos	CAM, EB, MFL	> 12	IFN-γ , rituximab	Relapse	(*6*)
F, 60	Laos	AMK, AZI/CAM, EB, INH, LFL/MFL, PZA, RMP	12	Rituximab	Cured	(*6*)
F, 45	Thailand	AMK, CAM, EB	8	None	Cured	Picque et al., 2012 (*11*
F, 40	Thailand	AZI/CAM, EB, LFL, RMP	Under treatment	None	Improved	Chaisathaphol et al., 2013 (*12*)
M, 50	China	NA	NA	NA	Cured	Chi et al., 2013 (*1*)
F, 47	China	NA	NA	NA	Persistent infection	(*1*)
M, 69	China	NA	NA	NA	Persistent infection	(*1*)
F, 47	China	NA	NA	NA	Persistent infection	(*1*)
M, 55	China	NA	NA	NA	Cured	(*1*)
M, 81	China	NA	NA	NA	Persistent infection	(*1*)
M, 61	China	NA	NA	NA	Cured	(*1*)
M, 84	China	NA	NA	NA	Cured	(*1*)
M, 44	China	NA	NA	NA	Cured	(*1*)
F, 59	China	NA	NA	NA	Persistent infection	(*1*)
M, 48	China	NA	NA	NA	Persistent infection	(*1*)
F, 48	China	NA	NA	NA	Persistent infection	(*1*)
F, 45	China	NA	NA	NA	Persistent infection	(*1*)
F, 72	China	NA	NA	NA	Died	(*1*)
F, 47	China	NA	NA	NA	NA	(*1*)
F, 47	China	NA	NA	NA	Persistent infection	(*1*)

Case-patient sex, age (y)	Origin	Baseline treatment regimen	Duration (months)	Immune therapies	Outcome	Reference
M, 60	China	NA	NA	NA	Persistent infection	(*1*)
M, 78	Japan	AZI, IMI, MFL, TM	NA	Rituximab	Relapse	Czaja et al., 2013 (*13*)
M, 66	Japan	AMK, CAM, EB, LVF, RMP	NA	None	Cured	Ishii et al., 2013 (*14*)
M, 53	Japan	EB, INH, RMP	NA	None	Died	Nei et al., 2013 (*15*)
F, 49	Laos	CAM, EB, RMP	NA	None	Died	Poulin et al., 2013 (*16*)
F, 64	Japan	CAM, EB, RFP	NA	None	NA	Yamaguchi et al., 2013 (*17*)
F, 47	Asia	Included LNZ	8.5	NA	Relapse	Chetchotisakd et al., 2014 (*18*)
M, 61	Asia	Included LNZ	35.5	NA	Cured	Browne et al., 2012 (*19*)
F, 15	Asia	Included LNZ	31	NA	Cured	(*19*)
F, 47	Asia	Included LNZ	24.5	NA	Relapse	(*19*)
M, 48	Asia	Included LNZ	25	NA	Persistent infection	(*19*)
F, 45	Asia	Included LNZ	126	NA	Relapse	(*19*)
F, 43	Asia	Included LNZ	59	NA	Relapse	(*19*)
F, 42	Asia	Included LNZ	49	NA	Died	(*19*)
F, 47	Asia	Included LNZ	104.5	NA	Cured	(*19*)
F, 45	Asia	Included LNZ	24	NA	Persistent infection	(*19*)
F, 57	Asia	Included LNZ	75.5	NA	Persistent infection	(*19*)
F, 48	Asia	Included LNZ	76	NA	Persistent infection	(*19*)
M, 63	Asia	Included LNZ	18.5	NA	Cured	(*19*)
M, 34	Asia	Included LNZ	33.5	NA	Persistent infection	(*19*)
F, 34	Asia	Included LNZ	21.5	NA	Cured	(*19*)
F, 39	United States	AZI, EB, LFL, RMP	Under treatment	None	Improved	O'Connell et al., 2014 (*20*)
F, 65	Germany	CAM, EMB, RMP	18	Rituximab	Persistent infection	Hanitsch et al., 2015 (*21*)
M, 65	Japan	CAM, EMB, RMP	36	NA	Cured	Hase et al., 2015 (*22*)
F, 65	Japan	CAM, EMB, KAN/LVX, RMP	24	NA	Relapse	Nishimura et al., 2015 (*23*)
M, 71	Cambodia	CAM, CIP, RMP	Under treatment	None	Improved	Otome et al., 2015 (*24*)
F, 50	Laos	AMK, AZI, EB, MFL, RIB	24	None	Cured	Present case

*AMK, amikacin; CAM, clarithromycin; EB, ethambutol; INH, isoniazide; PZA, pyrazinamide; NA, data not available; IFNg, interferon gamma; CFZ, clofazimine; RIB, rifabutin; LNZ, linezolid; MFL, moxifloxacin; TIG, tigecyclin; INH, isoniazide; AMX/CLV, amoxicillin/clavulanate; AZI, azithromycin; CFX, ciprofloxacin; MER, meropenem; RMP, rifampin; IvIg, intravenous immunoglobulins; SM, streptomycin; AZI, azithromycin; LFL, levofloxacin; IMI, imipenem; TM, tobramycin; LFL, levofloxacin; EMB, ethambutol; KAN, kanamycin; LVX, levofloxacin; CIP, ciprofloxacin.

References

1. Chi C-Y, Chu C-C, Liu J-P, Lin C-H, Ho M-W, Lo W-J, et al. Anti-IFN-γ autoantibodies in adults with disseminated nontuberculous mycobacterial infections are associated with HLA-DRB1*16:02 and HLA-DQB1*05:02 and the reactivation of latent varicella-zoster virus infection. Blood. 2013;121:1357–66. PubMed http://dx.doi.org/10.1182/blood-2012-08-452482

2. Döffinger R, Helbert MR, Barcenas-Morales G, Yang K, Dupuis S, Ceron-Gutierrez L, et al. Autoantibodies to interferon-γ in a patient with selective susceptibility to mycobacterial infection

and organ-specific autoimmunity. Clin Infect Dis. 2004;38:e10–4. PubMed http://dx.doi.org/10.1086/380453

3. Höflich C, Sabat R, Rosseau S, Temmesfeld B, Slevogt H, Döcke W-D, et al. Naturally occurring anti-IFN-γ autoantibody and severe infections with *Mycobacterium cheloneae* and *Burkholderia cocovenenans*. Blood. 2004;103:673–5. PubMed http://dx.doi.org/10.1182/blood-2003-04-1065

4. Kampmann B, Hemingway C, Stephens A, Davidson R, Goodsall A, Anderson S, et al. Acquired predisposition to mycobacterial disease due to autoantibodies to IFN-γ. J Clin Invest. 2005;115:2480–8. PubMed http://dx.doi.org/10.1172/JCI19316

5. Patel SY, Ding L, Brown MR, Lantz L, Gay T, Cohen S, et al. Anti-IFN- γ autoantibodies in disseminated nontuberculous mycobacterial infections. J Immunol Baltim Md 1950. 1 Oct 2005;175(7):4769-76. PubMed

6. Browne SK, Zaman R, Sampaio EP, Jutivorakool K, Rosen LB, Ding L, et al. Anti-CD20 (rituximab) therapy for anti-IFN-γ autoantibody-associated nontuberculous mycobacterial infection. Blood. 2012;119:3933–9. PubMed http://dx.doi.org/10.1182/blood-2011-12-395707

7. Tanaka Y, Hori T, Ito K, Fujita T, Ishikawa T, Uchiyama T. Disseminated *Mycobacterium avium* complex infection in a patient with autoantibody to interferon-γ. Intern Med. 2007;46:1005–9. PubMed http://dx.doi.org/10.2169/internalmedicine.46.6452

8. Baerlecken N, Jacobs R, Stoll M, Schmidt RE, Witte T. Recurrent, multifocal *Mycobacterium avium-intercellulare* infection in a patient with interferon-γ autoantibody. Clin Infect Dis. 2009;49:e76–8. PubMed http://dx.doi.org/10.1086/605581

9. Koya T, Tsubata C, Kagamu H, Koyama K, Hayashi M, Kuwabara K, et al. Anti-interferon-γ autoantibody in a patient with disseminated *Mycobacterium avium* complex. J Infect Chemother. 2009;15:118–22. PubMed http://dx.doi.org/10.1007/s10156-008-0662-8

10. Kampitak T, Suwanpimolkul G, Browne S, Suankratay C. Anti-interferon-γ autoantibody and opportunistic infections: case series and review of the literature. Infection. 2011;39:65–71. PubMed http://dx.doi.org/10.1007/s15010-010-0067-3

11. Picque J-B, Blot M, Binois R, Jeudy G, Simonet A-L, Cagnon J, et al. Recurrent atypical mycobacterial infections in the adult: think of autoantibodies against interferon-gamma [in French]! Rev Médecine Interne. 2012;33:103–6. http://dx.doi.org/10.1016/j.revmed.2011.11.012

12. Chaisathaphol T, Jitmuang A. Disseminated *Mycobacterium avium* and recurrent *Salmonella* group D infection in a patient with autoantibodies to interferon-gamma. Southeast Asian J Trop Med Public Health. 2013;44:460–7. PubMed

13. Czaja CA, Merkel PA, Chan ED, Lenz LL, Wolf ML, Alam R, et al. Rituximab as successful adjunct treatment in a patient with disseminated nontuberculous mycobacterial infection due to acquired anti-interferon-γ autoantibody. Clin Infect Dis. 2014;58:e115–8. PubMed http://dx.doi.org/10.1093/cid/cit809

14. Ishii T, Tamura A, Matsui H, Nagai H, Akagawa S, Hebisawa A, et al. Disseminated *Mycobacterium avium* complex infection in a patient carrying autoantibody to interferon-γ. J Infect Chemother. 2013;19:1152–7. PubMed http://dx.doi.org/10.1007/s10156-013-0572-2

15. Nei T, Okabe M, Mikami I, Koizumi Y, Mase H, Matsuda K, et al. A non-HIV case with disseminated *Mycobacterium kansasii* disease associated with strong neutralizing autoantibody to interferon-γ. Respir Med Case Rep. 2013;8:10–3. PubMed http://dx.doi.org/10.1016/j.mcr.2012.11.003

16. Poulin S, Corbeil C, Nguyen M, St-Denis A, Côté L, Le Deist F, et al. Fatal *Mycobacterium colombiense*/*cytomegalovirus* coinfection associated with acquired immunodeficiency due to autoantibodies against interferon gamma: a case report. BMC Infect Dis. 2013;13:24. PubMed http://dx.doi.org/10.1186/1471-2334-13-24

17. Yamaguchi E, Tanaka H, Fukuoka T, Ohbayashi Y, Sato M, Yokoi T. A case of disseminated nontuberculous mycobacteriosis and cerebellar toxoplasmosis with autoantibody to interferon-γ. Sarcoidosis Vasc Diffuse Lung Dis. 2013;30:312–6. PubMed

18. Chetchotisakd P, Anunnatsiri S. Linezolid in the treatment of disseminated nontuberculous mycobacterial infection in anti-interferon-gamma autoantibody-positive patients. Southeast Asian J Trop Med Public Health. 2014;45:1125–31. PubMed

19. Browne SK, Burbelo PD, Chetchotisakd P, Suputtamongkol Y, Kiertiburanakul S, Shaw PA, et al. Adult-onset immunodeficiency in Thailand and Taiwan. N Engl J Med. 2012;367:725–34. PubMed http://dx.doi.org/10.1056/NEJMoa1111160

20. O’Connell E, Rosen LB, LaRue RW, Fabre V, Melia MT, Auwaerter PG, et al. The first US domestic report of disseminated Mycobacterium avium complex and anti-interferon-γ autoantibodies. J Clin Immunol. 2014;34:928–32. PubMed http://dx.doi.org/10.1007/s10875-014-0073-9

21. Hanitsch LG, Löbel M, Müller-Redetzky H, Schürmann M, Suttorp N, Unterwalder N, et al. Late-onset disseminated *Mycobacterium avium intracellulare* complex infection (MAC), cerebral toxoplasmosis and *Salmonella* sepsis in a German caucasian patient with unusual anti-interferon-gamma IgG1 autoantibodies. J Clin Immunol. 2015;35:361–5. PubMed http://dx.doi.org/10.1007/s10875-015-0161-5

22. Hase I, Morimoto K, Sakagami T, Kazumi Y, Ishii Y, van Ingen J. Disseminated *Mycobacterium gordonae* and *Mycobacterium mantenii* infection with elevated anti-IFN-γ neutralizing autoantibodies. J Infect Chemother. 2015;21:468–72. PubMed http://dx.doi.org/10.1016/j.jiac.2015.02.003

23. Nishimura T, Fujita-Suzuki Y, Yonemaru M, Ohkusu K, Sakagami T, Carpenter SM, et al. Recurrence of disseminated *Mycobacterium avium* complex disease in a patient with anti-gamma interferon autoantibodies by reinfection. J Clin Microbiol. 2015;53:1436–8. PubMed http://dx.doi.org/10.1128/JCM.03339-14

24. Otome O, O'Reilly M, Lim L. Disseminated *Mycobacterium haemophilum* skeletal disease in a patient with interferon-gamma deficiency. Intern Med J. 2015;45:1073–6. PubMed http://dx.doi.org/10.1111/imj.12875
